# Surveillance, Diversity and Vegetative Compatibility Groups of *Fusarium oxysporum* f. sp. *vasinfectum* Collected in Cotton Fields in Australia (2017 to 2022)

**DOI:** 10.3390/pathogens11121537

**Published:** 2022-12-14

**Authors:** Duy P. Le, Chi P. T. Nguyen, Dinesh Kafle, Linda Scheikowski, Janelle Montgomery, Emma Lambeth, Amanda Thomas, Kieran O’Keeffe, Beth Shakeshaft, Alison Young, Andrew Mckay, Annabel Twine, Elsie Hudson, Rodney Jackson, Linda J. Smith

**Affiliations:** 1 New South Wales Department of Primary Industries, Narrabri, NSW 2390, Australia; 2 Queensland Department of Agriculture and Fisheries, Dutton Park, QLD 4102, Australia; 3 Queensland Department of Agriculture and Fisheries, Toowoomba, QLD 4350, Australia; 4 CottonInfo, Moree, NSW 2400, Australia; 5 CottonInfo, Wee Waa, NSW 2388, Australia; 6 CottonInfo, Warren, NSW 2824, Australia; 7 CottonInfo, Griffith, NSW 2680, Australia; 8 New South Wales Department of Primary Industries, Yanco, NSW 2703, Australia; 9 CottonInfo, St. George, QLD 4487, Australia; 10 CottonInfo, Dalby, QLD 4405, Australia; 11 Cotton Research and Development Corporation, Goondiwindi, QLD 4390, Australia

**Keywords:** cotton diseases, vascular wilt, vascular discolouration, *Fusarium* races, exotic pathogen

## Abstract

Cotton (*Gossypium hirsutum*) is a billion-dollar crop in regional New South Wales (NSW) and Queensland, Australia. Fusarium wilt (FW) caused by *Fusarium oxysporum* f. sp. *vasinfectum* (Fov) is an economically important disease. Initial disease losses of up to 90% when the disease was first detected resulted in fields being taken out of cotton production. The disease is now well-managed due to the adoption of highly resistant varieties. However, annual disease surveys recently revealed that the disease dynamic has changed in the past few seasons. With relatively mild and wet weather conditions during the 2021/22 growing season, FW was detected in eight surveyed valleys in NSW and Queensland, with the disease incidence as high as 44.5% and 98.5% in individual fields in early and late seasons, respectively. Fov is genetically distinct and evolved from local *Fusarium oxysporum* strains. Additionally, the pathogen was reported to evolve rapidly under continuous cotton cropping pressure. However, our knowledge of the genetic composition of the prevailing population is limited. Sequences of the translation elongation factor alpha 1 (TEF1) revealed that 94% of *Fusarium* isolates recovered from FW-infected cotton were clustered together with known Australian Fov and relatively distant related to overseas Fov races. All these isolates, except for nine, were further confirmed positive with a specific marker based on the Secreted in Xylem 6 (SIX6) effector gene. Vegetative compatibility group (VCG) analyses of 166 arbitrarily selected isolates revealed a predominance of VCG01111. There was only one detection of VCG01112 in the Border Rivers valley where it was first described. In this study, the exotic Californian Fov race 4 strain was not detected using a specific marker based on the unique *Tfo1* insertion in the phosphate (PHO) gene. This study indicated that the prevalence and abundance of Fov across NSW and Queensland in the past five seasons was probably independent of its genetic diversity.

## 1. Introduction

Cotton (*Gossypium hirsutum*) is an important crop in regional New South Wales (NSW) and Queensland, Australia generating approximately AUD 1.9 billion-dollar revenue annually [1]. Disease is a major constraint to sustainable and profitable production [2]. Annual disease surveys for quantitative assessments of cotton diseases have been continuously conducted in NSW since the 1983/84 cropping season [3] and in Queensland since 2002/03 (L. Smith, unpublished data). There has been a shift in the most prevalent disease over time. For example, bacterial blight caused by *Xanthomonas axonopodis* pv. *malvacearum* was the biggest constraint for Australian cotton production prior to the 1984 season. The incidence, however, was reduced sharply with the wide adoption of resistant varieties and has been undetected since the 1990/91 season [3]. Similarly, the incidence of Verticillium wilt (VW), caused by *Verticillium dahliae* dropped from over 16% in the 1989/90 season to below 3% in the 1996/97 season when resistant varieties comprised over 85% of the planting area in the 1996/97 season [3]. However, VW incidence has increased in the past five seasons and currently is the disease of most concern [2]. Fusarium wilt (FW) caused by *Fusarium oxysporum* f. sp. *vasinfectum* (Fov) increased exponentially between the first detection in NSW in 1994 and the 2002/03 season, after which new detections increased slowly [3]. In Queensland, FW was first discovered in fields on the Darling Downs in 1993, and the distribution of the disease since that time has been closely monitored [4]. By the year 2000, FW had spread to other Queensland cotton-growing regions [5]. In the 2008/09 season, FW was detected in 82% of crops inspected on the Darling Downs; however, the average incidence of FW was reduced compared to the previous three seasons. A similar trend was observed in other cotton-growing regions in Queensland. The observed decrease was attributed to later planting to avoid cooler spring conditions, widespread use of new, more resistant varieties, and wider use of the BION seed treatment in Queensland cotton production [6]. Current commercial varieties express a high level of resistance against Fov, with an F-rank above 113 for most varieties. F-rank is a rating system for FW resistance in cotton developed by cotton breeders where varieties are increasingly susceptible to Fov when the F-rank falls below 100 [7]. In a recent survey conducted by Crop Consultant Australia, only 2% of surveyed consultants reported an estimated loss of around 10% in association with FW [8]. This suggests that the FW disease has been well managed through the adoption of highly resistant varieties. Our surveillance enabled the continuation of disease prevalence and distribution to be captured over the past five seasons from 2017 to 2022, which is crucial for understanding disease dynamics.

Fusarium wilt of cotton caused by *Fusarium oxysporum* f. sp. *vasinfectum* was first identified in Alabama, USA, in 1892, and the disease is now widely detected in many cotton-growing countries across the globe [9]. Belonging to a *Fusarium oxysporum* species complex (FOSC), Fov collected across different geographic locations exhibited a high level of interspecific variation. Four Fov lineages were established based on multigene analyses of translation elongation factor alpha 1 (TEF1), β-tubulin and phosphate permase (PHO). Lineage I contained Fov isolates belonging to races 3 and 5. Lineage II contained Fov races 1, 2 and 6. Lineage III contained race 8 isolates. Lineage IV contained race 4 and 7 isolates [10]. Australian Fov is genetically distinct, forming an independent lineage [10,11]. Australian Fov originated from local *F. oxysporum* strains and evolved quickly [12]. However, our current knowledge of the genetics of the current Fov population is limited. Additionally, plant pathologists recently reported other *Fusarium* species cause wilt in cotton. For example, in a comprehensive survey in New Mexico, USA, species of *F. fujikuroi*, *F. proliferatum* and *F. solani* were reported to cause wilt disease of Pima cotton in the fields [13,14,15]. In Australia, changes in disease dynamics have been observed in the past five years, with disease incidence and severity increasing in some regions (D.P. Le, unpublished data). This could be due to changes in the pathogen population and diversity; hence, it is important to monitor and understand the prevailing Fov population structure. In this study, methodology included: (1) sequence of the TEF1 gene being one of the three most informative genes for delimiting *Fusarium* [16] for diversity study; (2) Australian biotype specific marker based on effector gene called Secreted in Xylem (SIX6) [17]; (3) Vegetative Compatibility Group (VCG) analysis using *nit* testers specific to Australian VCGs [11] to confirm the Australian Fov identification, as well as (4) Californian Fov race 4 marker based on the unique *Tfo1* insertion in the PHO gene [18] to monitor for a possible occurrence of exotic Californian Fov race 4 strains. Californian Fov race 4 is highly virulent and classified as nematode-independent [19]. This race has now spread to other USA states, i.e., New Mexico and Texas [20,21]. In Australia, Californian Fov race 4 is classified as a pathogen of high biosecurity concern.

In Australia, FW was first detected in Cecil Plains, Queensland, in 1993 and Boggabilla, NSW, in 1994 [4] and subsequently detected in many other regions [3,22]. Australian Fov isolates from Cecil Plains were designated to vegetative compatibility group 01111 (VCG01111), which was also detected in many other regions [22]. On the other hand, isolates from Boggabilla belonged to VCG01112, which was largely restricted to the region [22]. In 2005, an isolate of Fov recovered from diseased cotton in the Mungindi region of NSW that was not vegetatively compatible with either Australian VCG testers was confirmed to be pathogenic on cotton in a glasshouse bioassay. The isolate was genetically different from Australian representatives of VCG01111 and VCG01112 and, over the IGS region, was more similar to VCG01112 than to VCG01111 (S. Van Brunschot, per. comm.). Further isolates were recovered from diseased cotton over two seasons from the same field, which were clearly included in the lineage A group but were considerably different from both VCGs 01111 and 01112 [23,24]. It was considered that these isolates may represent a third form of Fov and possibly a new VCG. However, recovery of the new form was unsuccessful after 2008. Wang et al. [22] also found a genetically distant subgroup within the VCG01111, raising the possibility of new VCGs forming again. VCG is a common marker for genetic studies in ascomycota, including *Fusarium*. Currently, there are 23 recognised and a number of undetermined VCGs within Fov [25]. Within Fov, relationships between VCGs and virulence (races) and molecular diversity based on RADP (random amplified polymorphic DNA) and RFLP (restriction fragment length polymorphism) analyses of rDNA and mtDNA were relatively complex [26,27]. Fernandez et al. [27] reinforced for consideration VCGs as independently evolving lineages within Fov. Therefore, in this study, VCG analysis was employed as an additional marker for the identification of Australian Fov, as well as for monitoring the population distribution across major cotton growing in Australia.

## 2. Materials and Methods

### 2.1. Disease Surveillance

Commercial cotton crops were inspected each season between October and December for early season assessment and between the following February to May for late season assessment. Early-season disease surveys were conducted 3–6 weeks after sowing and before the first irrigation. Late-season surveys were conducted after the final irrigation but before defoliation. A standardised survey method was used to assess fields for Fusarium wilt incidence. For example, a total of 200 (or 300) plants from each field were sampled using a step-point method across two (or three) transects. The first sampling was taken 50 m into the field at the tail drain end, where a GPS coordinate was auto-logged using the mobile app Fulcrum^©^. The second sampling point was reached by walking across 10 rows, then up the tenth row 20 m. This pattern was repeated until 10 sampling sites had been surveyed. The second transect and third, if conducted, also entered at the tail drain as described for transect 1 but spread along the width of the field.

**Figure 1:**
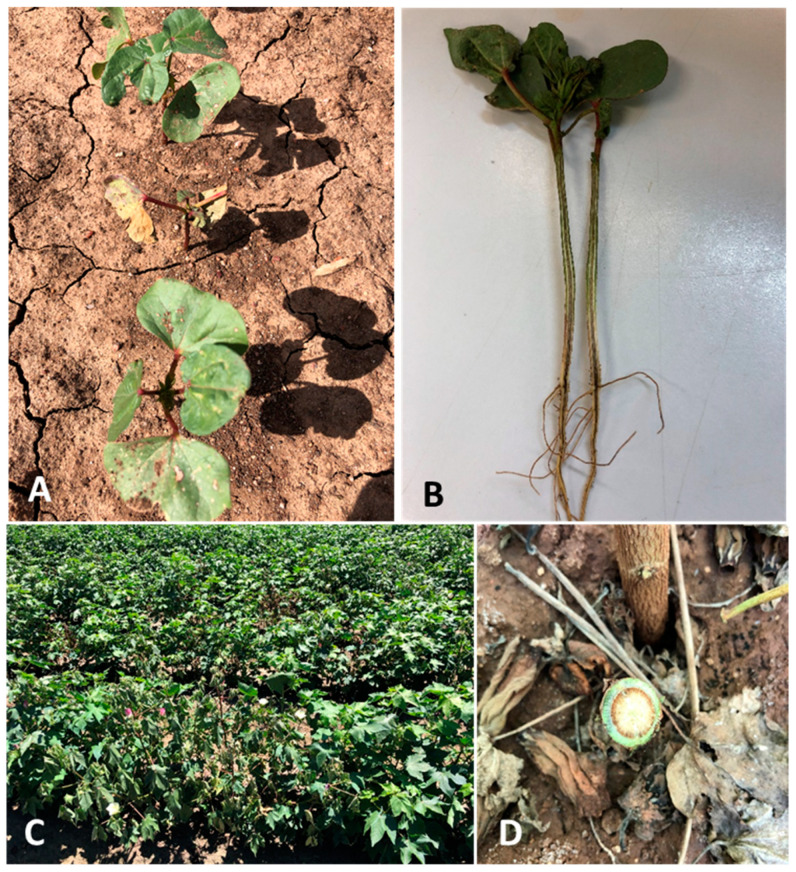
Field symptoms of Fusarium wilt expressed in cotton seedlings and mature cotton. (**A**) stunted seedling with cotyledon yellowing and necrosis; (**B**) profound vascular discolouration was observed upon splitting of the stem; (**C**) wilted cotton occurred in single or group; (**D**) circular vascular discolouration was characteristic of Fusarium wilt.

During early-season surveys, at each of the 10 sampling points, 10 plants were visually examined for external symptoms of Fusarium wilt, such as wilting, leaf yellowing and necrosis and plant stunting or death (Figure 1A). The 10 plants were then carefully removed from soil using an asparagus knife or trowel and examined internally for vascular discolouration (Figure 1B). The number of plants expressing Fusarium wilt symptoms was recorded, and plant samples were collected for pathogen isolation and confirmation.

During late-season surveys, sampling was similar to that described for early-season surveys. External symptoms of disease, such as plant wilting (Figure 1C), may be observed; however, in high F-ranked varieties, external symptoms were not always evident in diseased plants. Hence, stems of the 10 plants were cut near the base and assessed for vascular discolouration (Figure 1D), and diseased stem samples were collected for pathogen isolation.

### 2.2. Sampling and Pathogen Isolation

FW-suspected diseased cotton, including seedlings and mature stem pieces (approx. 10–15 cm in length), were sampled for *Fusarium* isolation and confirmation of Fov. Where possible, at least three FW-suspected seedlings or stem pieces were sampled from each of the surveyed fields. These samples were double-bagged inside a paper bag and an outer zip-lock plastic bag, stored in a portable cooler away from direct sunlight during the survey trips and transferred to a 4 °C cold room after each trip until further processing.

To isolate the putative *Fusarium* from vascular tissue, diseased samples were first excised into sections (1–2 cm long) and outer bark peeled off. Under aseptic conditions, the vascular tissue was sprayed and left for 30 s with 70% ethanol for surface decontamination, followed by blotting dry with paper towel. Discoloured vascular tissues were thinly sliced using a surgical scalpel and embedded into potato dextrose agar (PDA Difco) amended with 100 ppm streptomycin sulfate (Sigma Aldrich, St Louis, MO, USA) (sPDA) contained in 90 mm dia. Petri plates. The plates were then sealed with parafilm and incubated at 25 °C in darkness for 2–3 days [28]. Putative *Fusarium* colonies emerging from the vascular tissues were individually sub-cultured onto new sPDA plates, and single spore cultures were established. Pure cultures were then transferred onto 2% water agar (WA) and incubated at 25 °C in darkness for at least a week before small plugs (0.5 cm^2^) were excised from the active growing cultures and submerged in sterile water contained in 2 mL sterile vials for long term storage at room temperature.

### 2.3. DNA Extraction

A Wizard^®^ Genomic DNA Purification Kit (Promega, Sydney, Australia) was used following the manufacturer’s protocol to obtain genomic DNA. However, the protocol was slightly modified as per described by Le et al. [28] to suit our laboratory conditions. Mycelia (10–100 mg) was scraped off culture plates using a surgical scalpel and transferred into a 1.5 mL tight-lock Eppendorf tube. Fifty µL Nuclei Lysis Solution was added along with two steel beads (3.3 mm dia.) to each tube. The tubes were then placed on a tissue lyser (Retsch^®^ MM300) and shaken for 1 min at a frequency of 28 times per second to macerate the mycelia. Once macerated, another 450 µL Nuclei Lysis Solution was then added to each of the tubes, which were vortexed to homogenicity and incubated in a water bath pre-set at 65 °C for 30 min. After incubation, 3 µL RNase A Solution was added to each of the tubes and followed by another incubation at 37 °C for 15 min. After reaching ambient temperature, 200 µL Protein Precipitation Solution was added and vortexed vigorously to homogenicity. The tubes were then centrifuged at 13,000 rpm for 5 min. The supernatants were carefully transferred to new 1.5 mL Eppendorf tubes containing 600 µL room temperature isopropanol. The tubes were gently inverted and centrifuged at 13,000 rpm for 1 min. The supernatants were carefully decanted, and the visible DNA pellets were washed twice with 70% room-temperature ethanol. The DNA pellets were then air-dried under a fume hood for 30–45 min, rehydrated with 50–200 µL DNA Rehydration Solution depending on the size of the pellets, and incubated at 65 °C for 1 h. The DNA solutions were then stored at −20 °C until use.

### 2.4. PCR Amplification for the TEF1

The primer pair EF1-728F 5′-CATCGAGAAGTTCGAGAAGG-3′ and EF2 5′-GGA(G/A)GTACCAGT(G/C)ATCATGTT-3′ was used for amplification of the TEF1 region as per descriptions by Le et al. [29]. GoTaq^®^ G2 Green Master Mix (Promega, Sydney, Australia) was used for all PCR amplifications. Each PCR mix contained: 12 µL of Green Master Mix, 6 µL of DNase-free water, 1 µL of 10 mM primer mix and 1 µL of DNA template. DNase-free water was included as a negative (no-template) control. PCR cycling conditions were as follows: initial denaturation at 94 °C for 5 min followed by 35 cycles of 30 s at 94 °C, 30 s at 52 °C and 90 s at 72 °C, with a final elongation step of 7 min at 72 °C. Integrity of amplified PCR products was confirmed by electrophoresis. The products were run at 100 V in a 1.5% agarose gel pre-stained with GelRed (GeneTargetSolutions, Dural, Australia) for 45 min and visualised under UV light using a UVIDOC HD6 (UVITEC, Cambridge, UK). All PCR products were purified and sequenced by Macrogen Inc., Seoul, Republic of Korea. All sequences retrieved from both forward and reverse primers were manually adjusted for their consensus sequences. Multi-sequence alignments of the TEF1 and maximum likelihood phylogenetic analyses using Tamura-Nei model with 1000 bootstrap replicates were performed with MEGA 7 [30]. Reference sequences were also downloaded from GenBank and included in phylogenetic analyses.

### 2.5. Australian Fov Detection

The primer pair SIX6-R1 5′-CAAGACCAGGTGTAGGCATT-3′ and FovSIX6-F2 5′-CTTCACGGCAGACCCG-3′, developed by Chakrabarti et al. [17] was used for specific detection and confirmation of the Australian Fov biotypes. The PCR amplification and the integrity of the amplicons were carried out as described previously. The PCR cycling conditions were as follows: initial denaturation at 94 °C for 2 min followed by 30 cycles of 94 °C for 30 s, 55 °C for 30 s, 72 °C for 1 min and final extension at 72 °C for 5 min. A number of arbitrarily selected isolates/amplicons were sent to Macrogen for sequencing.

### 2.6. Californian Fov Race 4 Detection

Putative *Fusarium* isolates that failed to be amplified with the specific SIX6 primers were further subjected to another round of PCR amplifications with the primer pair FovP-F 5′-GGCCGATATTGTCGGTCGTA-3′ and FovT-R 5′-ATCTGTCTTTCGTCGGCAAT-3′ [18]. This primer pair was designed based on the unique *Tfo1* insertion in the PHO gene, which is presented only in Californian Fov race 4 isolates. The PCR amplification and the integrity of the amplicons were carried out as described previously. The PCR cycling conditions were as follows: initial denaturation at 94 °C for 2 min followed by 35 cycles of 94 °C for 30 s, 58 °C for 30 s, 72 °C for 2 min, and final extension at 72 °C for 5 min.

### 2.7. VCG Determination

The vegetative compatibility of *F. oxysporum* isolates derived from diseased cotton stems, and reference Fov strains were tested using the method described by Puhalla [31] and Correll et al. [32]. A brief description is provided below.

### 2.8. Reference Isolates of Australian F. oxysporum f. sp. vasinfectum

Isolates 24300-2 and 24350-2 of VCG 01111 and isolate G14 of VCG 01112 (Queensland Department of Agriculture and Fisheries, Dutton Park, Australia) were used as references of pathogenic Australian Fov in this study.

### 2.9. Generating Nitrate Non-Utilising (nit) Mutants

For each isolate, nitrate non-utilizing (*nit*) mutants were generated by streaking Fov culture onto two potassium chlorate (KClO_3_) supplemented PDA (KPS) plates. They were incubated at room temperature for 7–14 days. A normal fast-growing mycelial sector (resistant sector) of an Fov isolate on the medium emerging from the restricted colony was sub-cultured from the advancing edge of the sector onto minimal medium (MM; [33]) for *nit*1 mutant identification. True *nit* mutants are not able to reduce the nitrate in the medium, and characteristically sparse, nitrogen-deficient growth will result. If the resulting growth was not sparse on MM, the culture was discarded and not used in VCG tests.

### 2.10. Pairing nit Mutants in VCG Tests

A small (2 mm^2^) piece of colonised agar from a culture of a *nit*M mutant of known VCG was placed in the centre of a plate of MM. Similar small pieces of culture of the *nit* mutants that were generated from the isolate of unknown VCG were then placed at least 10 to 15 mm away from the piece of *nit*M culture around the edge of the plate. Plates were incubated at room temperature for two weeks and checked every two days during this time for the formation of heterokaryon growth. Heterokaryon formation was identified by wild-type growth.

## 3. Results

### 3.1. Surveillance

A total of 527 fields across NSW and Queensland were visited for comprehensive in-field disease surveys during the early cropping seasons between 2017 and 2022 (Table 1). During the course of this study, FW on seedlings was not detected prior to the 2020/21 season in north to southwest valleys in NSW. FW was only detected on seedlings in the Gwydir valley (northwest NSW) in the last two seasons between 2020 and 2022. Two FW-infested fields were detected outside of the surveyed transects in the 2020/21 season. Eight FW fields were detected early season of 2021/22 in the Gwydir Valley, where the mean incidence in the FW-infested fields was as high as 5.8% (Table 1). In comparison, in Queensland, FW was detected during early-season surveys in all regions except Emerald, during all five seasons where surveys were conducted. The mean disease incidence early season ranged from 9 to 13.8% and was highest in the 2021/22 season across Theodore, St George and Darling Downs valleys compared to the previous four seasons. St George had fields with FW incidence as high as 26%, 30% and 26.5% in the 2017/18, 2018/19 and 2021/22 cropping seasons, respectively. The field with the highest FW incidence detected (44.5%) was on the Darling Downs in the 2021/22 season (Table 1). Fields detected with a high incidence of FW early season generally had a higher disease incidence detected late season (data not presented).

On the other hand, 82 fields in NSW were observed during the late-season surveys to have in-field FW symptoms, and the pathogen was confirmed with isolation and PCR amplification of the SIX6 for Australian Fov. The only exception was a field in the Namoi in the 2017/18 season, where the in-field symptoms were similar to those of FW, but upon specific PCR confirmation with the SIX6 marker, we failed to amplify *Fusarium* isolates recovered from these FW-like samples (Table 2). Another addition of 32 fields where the FW was detected outside of the survey transects. In total, 114 FW fields across the six valleys were detected during late-season survey in the past five cropping seasons between 2017 to 2022.

The mean disease incidence of FW during late-season surveys ranged from 0.5 to 39.4% (Table 2). In NSW, mean disease incidence in the Gwydir Valley was consistently higher than that recorded in other Valleys, and indeed had the highest recorded disease incidence of all NSW and Queensland surveyed regions since the 2019/20 season (Table 2). The mean disease incidence in FW-infested fields in the Gwydir in the 2021/22 season was up to 39.4%. More concerningly, a number of fields in the Gwydir had an FW incidence of up to 98.5%, and significant yield loss was also reported in the last two seasons (unpresented data). Meanwhile, the FW incidence in other valleys remained well below 5%, except for the two Macquarie fields in the 2018/19 season, where the mean incidence was at 9.8% (Table 2).

A total of 195 fields were surveyed during this study in Queensland for the late season between 2017 and 2022 (Table 1). Of these, 79 fields (41%) were determined late season to be infested with FW, confirmed by pathogen isolation and VCG analyses. The mean disease incidence late season ranged from 1 to 33% and was highest in the 2021/22 season across all valleys compared to the previous four seasons. In Emerald, for the three seasons surveyed, only one field in the 2021/22 season was detected with FW and at a low incidence. In Theodore, the mean disease incidence and number of fields detected with FW were low, except for one field with a disease incidence of 33% and 37% in 2018/19 and 2021/22, respectively. Further south in the Darling Downs and St George region, FW was detected every season in each valley ranging from 40–75% of fields surveyed. A broad range of disease incidences were detected, from as low as 0.5% to extremely high at 97% (Table 2).

### 3.2. TEF1-Based Diversity

**Figure 2:**
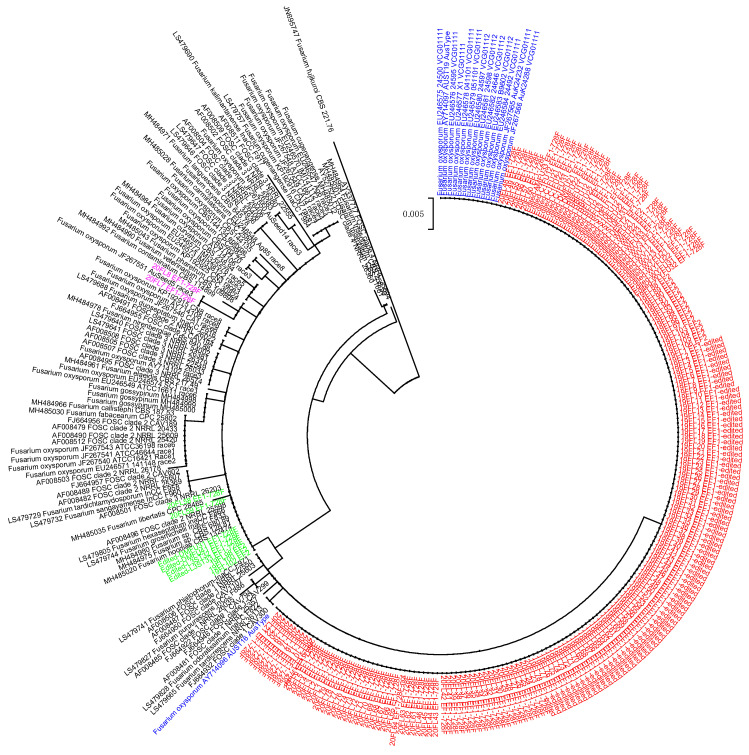
ML tree constructed based on sequences retrieved from TEF1 gene showing the relationship between Australian *F. oxysporum* recovered in this study (coloured text labels) and other GenBank references. Red (only 200 representative isolates were included in the tree) and blue text labels are for prevailing and referencing GenBank Australian Fov, respectively. Pink and green text labels are for *F. oxysporum* recovered from FW-cotton in this study, which were closely related to referencing isolates in lineage I and II, respectively.

A total of 412 arbitrarily selected isolates were subjected to PCR amplification and sequencing using the EF1-728F/EF2 primer pair targeting the TEF1 gene (Table S1). We successfully amplified and retrieved sequences of 389 isolates. Of which, 366 isolates accounting for 94% of the sequenced collection were well-clustered together in a maximum likelihood analysis and to those previously identified as Australian Fov, which was well separated from other known Fov races (Figure 2). All these, except for nine isolates, were further confirmed with the specific Australian Fov SIX6 marker. Two isolates were clustered with Fov race 3, which belonged to lineage I as defined by Skovgaard et al. [10]. Twenty-one isolates belonged to lineage II. Of these 23 isolates, 16 tested negatively against the specific Australian Fov SIX6 marker, while the remaining seven isolates tested positive (Table S1).

### 3.3. Australian Fov Identification

A total of 823 (i.e., 743 from NSW and 80 from Queensland) putative FOSC isolates were subjected to further confirmation for Australian Fov using the specific SIX6 primers. Of these, 675 NSW isolates (90.8%) (Table 3) and all 80 Queensland isolates were identified as Australian Fov (Table S1). Most Fov isolates were recovered from the Gwydir valley, which was highly associated with the level of FW disease detected in the valley. In NSW, Fov was only detected on seedlings in the last two seasons between 2020 to 2022. Prior to the 2020/21 season, no Fov was detected in the Namoi, a neighbouring valley of the Gwydir, though the FW-like symptoms were observed in one field in the 2017/18 season (Table 2), and corresponding FOSC isolates were recovered (Table 3). In other valleys, the number of confirmed Fov isolates highly fluctuated among seasons.

**Figure 3:**
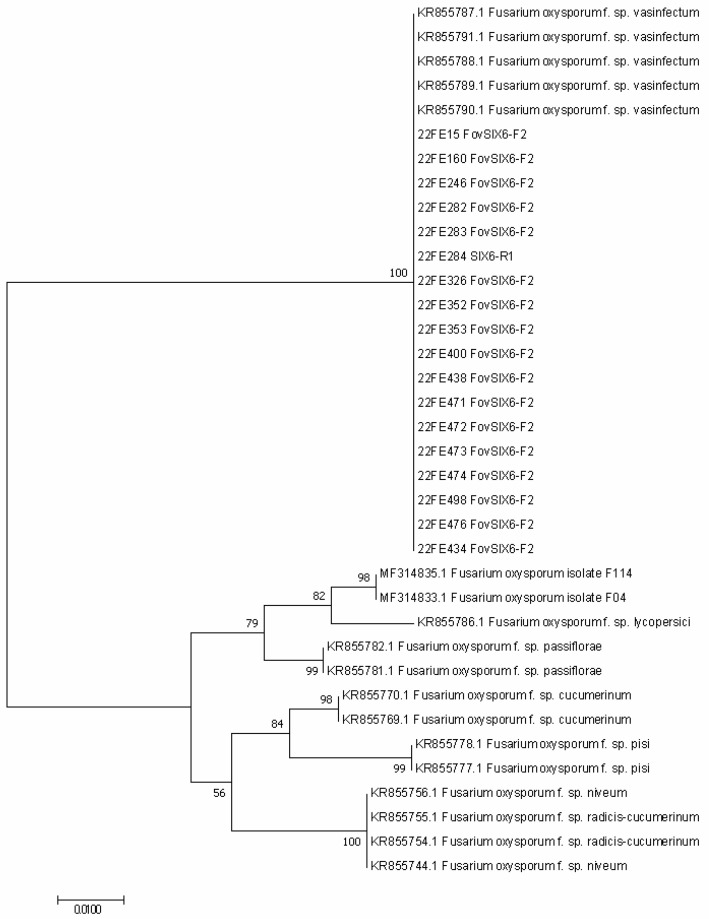
ML tree constructed based on sequences retrieved from SIX6 gene showing the relationship between Australian *F. oxysporum* recovered in this study (22FE isolates) and other GenBank references.

The SIX6 sequence data were also obtained for a total of 18 arbitrarily selected isolates, which were further subjected to pairwise and ML analyses. In the pairwise analysis, the 18 sequences were identical to each other. In the ML analysis, which included 18 additional reference sequences retrieved from GenBank, all 18 isolates in this study were clustered together with reference Australian Fov isolates and relatively distant from other formae speciales (Figure 3).

### 3.4. Californian Race 4 Detection

Isolates that belonged to FOSC based on the TEF1 sequences that were unsuccessfully amplified with the Australian Fov SIX6 marker were further subjected to another round of PCR for detection of the exotic Californian Fov race 4. To date, no Californian Fov race 4 has been detected in Australia.

### 3.5. VCGs

A total of 166 Fov isolates (i.e., 85 from NSW and 81 from Queensland) arbitrarily selected from the collection were subjected to VCG analyses (Table S1). VCGs were successfully determined for 149 isolates, accounting for 90%. Seventeen isolates from the NSW collection did not form a heterokaryon with the testers and were, therefore, not vegetatively compatible. Of the 149 isolates, all but one isolate recovered from the Border Rivers field was determined as VCG01111.

## 4. Discussion

This study provided comprehensive knowledge on the prevalence, distribution and dynamics of FW in NSW and Queensland, which is crucial for the identification of any new races. It was clear that the disease is well established in many surveyed fields in some valleys, i.e., Darling Downs and the Border Rivers, where the disease was first detected as well as in nearby valleys such as the Gwydir. Though the disease has spread to many other valleys, the pathogen is not well-established in Central Queensland. Wang et al. [35] found that the optimum temperature range for Fov in Australia was 18 °C to 23 °C with little development of symptoms above 23 °C. The authors suggested that suppression of the full symptom expression at relatively high temperatures (>33 °C) might have some epidemiological significance. Even the most susceptible cultivar Siokra 1–4 was less susceptible at higher temperatures. Cotton is a summer crop, and the further north the growing area (such as Central Queensland), the earlier the season can start with a wider growing window due to the warmer climate. Given this, the difference between the relative growth rates of pathogen and host may be one of the explanations for the influence of temperature on the disease and the limited spread of FW in Central Queensland compared to the cooler southern regions.

Fov recovered from FW-infected cotton in NSW and Queensland was highly similar. Approximately 94% of the TEF-sequenced isolates were clustered and formed a unique group within the FOSC. Similarly, a small group of arbitrarily selected isolates (*n* = 18) was also identical in their SIX6 sequences. Additionally, VCG analyses revealed a single VCG01111 in all but one field that was widely and dominantly distributed across NSW and Queensland, though there were some undetermined VCG isolates. In a previous report, Le et al. [29] revealed a high degree of diversity of *Fusarium*, especially within FOSC that recovered from atypical symptoms of FW on seedlings. However, only 21.5% of the collection was grouped together with Australian Fov [29]. The lack of diversity of *Fusarium* recovered from typical FW-infected cotton, especially within the Australian Fov, indicates that the pathogen is highly adapted to commercial cotton, which may also share a similar resistant background (only Upland cotton, *Gossypium hirsutum* is now grown in commercial fields). Though Australian Fov was deemed to have a fast evolution [22], the highly uniform population, based on analyses of TEF1 and SIX6 sequences and VCG, may suggest that this is not the case, despite the industry moving from cultivars that were highly susceptible to Fov to cultivars that now have much higher levels of resistance. Due to the unique TEF1-based position of Australian Fov in the ML analysis, this TEF1 gene fragment remains a reliable marker for the identification of Australian Fov. However, there was a small number of isolates (*n* = 9) that were clustered together with the Australian Fov that did not yield the specific SIX6 amplicon. Australian Fov is a true wilt pathogen causing vascular discolouration [4]. The absence of SIX6 amplicon in some isolates may indicate that they are not a true wilt pathogen. The 21.5% of the collection, equivalent to 34 FOSC isolates clustered with the Australian Fov reported by Le et al. [29], were subsequently tested negative against the SIX6 marker (D.P. Le, unpublished data). It is suggested that both TEF1 and SIX6 markers are greatly complemented in the genetic study and identification of Australian Fov. The lack of SIX6, which is Secreted in Xylem effector gene, in some isolates suggests that they might be a vascular incompetent pathogen, which was reported in some Fov isolates [36]. Therefore, this warrants a further pathogenicity test for these isolates.

The presence of SIX genes is a good prediction of Fo virulence; however, some non-pathogenic isolates also carry some fewer conservative SIX genes in comparison to their related pathogenic ones [37]. Australian Fov carried a combination of SIX6, SIX11, SIX13 and SIX14 genes, while Fov race 7 isolate NRRL25433 carried a combination of two SIX genes, i.e., SIX9-G1 and SIX13 [38]. According to Gawehns et al. [39], SIX6 was classified as a genuine effector gene, which plays a crucial role in the pathogenicity of *Fusarium oxysporum.* The presence of SIX6 in seven isolates that were closely related to FOSC lineage II determined by TEF1 phylogeny may indicate an occurrence of a new genotype but warrants further pathogenicity characterisation of these isolates. Interestingly, Rocha et al. [40] found two out of 115 isolates that were recovered from natural soil of minimal disturbance were positive for the SIX6 gene. This finding allowed them to reinforce the local origin of Australian Fov [40]. Most SIX genes are unique to *F. oxysporum*, but SIX6 homologues were also reported in other *Fusarium* spp. and some *Colletotrichum* spp. [38,39]. Therefore, a full SIX gene profile of these seven isolates is also desirable for further research.

Wang et al. [12] found that 81 Australian Fov isolates recovered from FW-cotton and included in their study were well clustered together and relatively distant from other overseas isolates (race 1–8 isolates). In our study, 23 *F. oxysporum* recovered from FW-cotton did not belong to the unique Australian Fov cluster. They also lacked a specific SIX6 marker and were not determined for their VCGs. Although *F. oxysporum* isolates closely related to Fov race 1–8 were recovered from soil and atypical FW symptoms seedlings, this appears to be the first time Fov isolates other than Australian biotypes have been detected from FW-diseased cotton. They could be new genotypes associated with Australian cotton. Zhu et al. [13,14,15] reported *Fusarium* spp. other than *F. oxysporum* that caused FW of cotton in the USA. Therefore, further characterisation of these isolates is warranted. Unlike Australian Fov biotypes, other Fov races, except for Californian race 4 require interaction with nematodes for infection [19]. The southern root-knot nematode, *Meloidogyne incognita*, has been reported as the most important nematode in association with Fusarium wilt of cotton [34,41,42]. In Australia, *M. incognita* has only been reported on cotton in one field each in Ayr (Northern Australia) and Theodore (Central Queensland) [43]. There have also been reports that the interaction between Fov and reniform nematode (*Rotylenchulus reniformis*) increases the intensity of Fusarium wilt of cotton [44]. Reniform has only been detected in Central Queensland on cotton in Australia [43]. Therefore, the risk of these Fov races becoming established on Australian cotton could be minimal.

Wang et al. [22] and Smith [23,24] observed that the VCG population shifted within fields and regions, but this was not the case for Australian isolates examined in this study, which was dominated by VCG01111. Only one VCG011112 isolate was detected in the fields where it was first detected. This may be due to the weak adaptability of VCG01112 to a broad range of geographic locations since host-resistant responses against the two VCGs appeared the same in bioassays (W. Stiller, per. comm.). VCG analyses are expensive, laborious and time-consuming; hence, only selected isolates were subjected to the analysis. Additionally, due to a limited number of testers available, 17 isolates were not determined for their VCG. Of these, 10 were characterised as Australian biotypes using TEF1 and SIX markers. Therefore, further characterisation of these isolates will be warranted to provide better insights into the existing Fov population.

To date, the Californian Fov race 4 remains undetected in Australia. This is an exotic isolate of the Australian cotton industry and is a high biosecurity concern in Australia. Californian Fov race 4 was first detected in California, causing severe wilt of Pima cotton [19], but now it is detected in many fields in New Mexico and Texas [20,21]. Californian Fov race 4 is highly virulent and does not require nematode infestation for infection. The pathogen is pathogenic to both Pima and Upland cotton though it was less virulent than Upland cotton [19,25]. The pathogen is soilborne; however, also known to be seedborne [45]. The Australian cotton industry is self-sufficient in seed production, which adheres to a seed production protocol to prevent the dispersal of Fov as seedborne inoculum (L. Smith, per. comm.). Additionally, only Upland varieties are grown in commercial fields. Therefore, it should be considered a low risk of introduction and establishment in Australian cotton fields. However, we should remain highly vigilant since a soilborne pathogen like Californian Fov race 4, if introduced, could result in long-lasting detrimental impacts on the industry. The most cost-effective management of the Californian Fov race 4 relies on breeding success. Some Pima cultivars were identified as having a high level of tolerance to the Californian Fov race 4, while such tolerant levels have not been identified in Upland cultivars [46]. Therefore, a continuation of the current surveillance program will be warranted.

In summary, FW has been established and is widely distributed across many cotton-growing valleys in Australia. The disease remains a major yield-limiting factor in many fields and is caused by a highly uniform pathogen. This suggests that the soilborne Fov has been introduced across cotton-growing valleys from its original source. Therefore, a better farm hygiene practice could assist in minimising the further spread of the pathogen. This also suggests that the increase in disease incidence does not appear to be due to a changing Fov population as determined by TEF1, SIX6 and VCG markers. The host crop, environmental conditions and cropping practices may have had a major impact on Fusarium wilt development. However, it is suggested that we should continue to monitor the Fov diversity and evolution since the pathogen population of *Fusarium* can be resolved better with amplified fragment length polymorphisms (AFLPs) [22], inter-simple sequence repeat (ISSR) [47] and genotyping-by-sequencing [48].

## Figures and Tables

**Table 1:** Summary of FW-infested fields per total number of surveyed fields and FW incidence recorded during early-season surveys from 2017 to 2021 in ten cotton-growing valleys in NSW and Queensland, Australia.

Valleys ^1^	Season 2017/18		Season 2018/19		Season 2019/20		Season 20/21		Season 21/22	
	FW Fields ^2^	Incidence (%) ^3^	FW Fields	Incidence (%)	FW Fields	Incidence (%)	FW Fields	Incidence (%)	FW Fields	Incidence (%)
**Emerald**	0/7	0	0/11	0	na	na	na	na	0/12	0
**Theodore**	1/11	1	1/11	2.5	na	na	1/14	4.7	5/18	2 (0.5–5)
**St George**	7/10	6 (1.5–26)	4/8	10.8 (3–30)	4/7	2.3 (0.5–6)	3/14	0.9 (0.5–2.3)	2/5	13.8 (1–26.5)
**Darling Downs**	1/11	5.5	10/17	4.7 (0.5–13.5)	5/18	3.1 (0.3–9)	8/20	4 (0.7–12.3)	11/13	7.1 (0.5–44.5)
**Border Rivers**	na	na	1/13	0.5	1/5	2.5	3/13	1.3 (0.3–3)	7/14	1.5 (0.5–4)
**Gwydir**	0/8	0	0/13	0	0/10	0	0 [34]/11	Detected	8 [34]/19	5.8 (0.3–20.5)
**Namoi**	0/20	0	0/18	0	0/10	0	0/10	0	0/20	0
**Macquarie**	0/10	0	0/10	0	0/6	0	0/7	0	0/13	0
**Lachlan**	0/8	0	0/9	0	0/8	0	0/7	0	0/5	0
**Murrumbidgee**	0/13	0	0/14	0	0/8	0	0/11	0	0/9	0

^1^ These ten valleys extended over a large geographic range of Australia, from Central Queensland (Emerald and Theodore) to the Border Rivers Region in southern Queensland and north-eastern New South Wales, and in NSW from northwest (Gwydir) to southwest (Murrumbidgee). ^2^ Number of FW-infested fields per total number of formally surveyed fields recorded during formal transect walks as per description in the materials and methods, while number in the box brackets indicate the number of FW-infested fields that were detected outside of the formally surveyed transects. ^3^ Mean incidence (%) recorded in surveyed transects in FW-infested fields. Where the FW was detected outside of the surveyed transects, it was indicated as ‘Detected’. In fields where Fusarium wilt was detected, the range of disease incidence from lowest to highest is in parentheses. na: data were not available since no formal survey was conducted.

**Table 2:** Summary of FW-infested fields per total number of surveyed fields and FW incidence recorded during late-season surveys from 2018 to 2022 in ten cotton-growing valleys in NSW and Queensland, Australia.

Valleys ^1^	Season 17/18		Season 18/19		Season 19/20		Season 20/21		Season 21/22	
	FW Fields ^2^	Incidence ^3^ (%)	FW Fields	Incidence (%)	FW Fields	Incidence (%)	FW Fields	Incidence (%)	FW Fields	Incidence (%)
**Emerald**	0/8	0	0/4	0	na	na	na	na	1/10	4.5
**Theodore**	3/10	5.7 (2–12)	1/10	33	5/19	4.3 (0.5–10)	0/7	0	2/10	20 (3–37)
**St George**	7/10	15 (0.5–46)	6/8	18 (0.5–58)	3/6	1.3 (0.5–2)	4/11	2.3 (0.5–3.5)	9/16	27.7 (0.5–97)
**Darling Downs**	5/10	5.2 (0.5–20)	11/18	4.3 (0.3–21)	6/17	4.9 (0.5–10)	6/8	4 (0.5–14)	10/13	15.9 (1.5–92)
**Border Rivers**	7/9	18 (1–60)	6/15	3.5 (0.5–10)	2/5	1 (0.5–1.5)	5/12	8.4 (0.5–20)	10/14	18 (3.3–61)
**Gwydir**	4 (3)/8	10.7 (0.5–50)	7 [34]/13	12.9 (0.5–68)	7 [34]/10	6.14 (1–39)	9 (10)/11	25.5 (1.5–96)	16 (1)/19	39.4 (0.5–98.5)
**Namoi**	0/20	0	0/18	0	0/10	0	0 (1)/10	Detected	0 [34]/20	Detected
**Macquarie**	1 (1)/10	0.7	2 (3)/10	9.8 (3.5–14)	0/6	0	0/7	0	0 (1)/13	Detected
**Lachlan**	0/8	0	0/9	0	1 [34]/8	0.5	1/7	1	0/5	0
**Murrumbidgee**	0/13	0	1 (1)/14	0.5	1/8	0.5	2 (1)/11	2.5 (1–4)	0/9	0

^1^ These ten valleys extended over a large geographic range of Australia, from central Queensland (Emerald and Theodore) to the Border Rivers Region in southern Queensland and north-eastern New South Wales, and in NSW from northwest (Gwydir) to southwest (Murrumbidgee). ^2^ Number of FW-infested fields per total number of formally surveyed fields recorded during formal transect walks as per described in the materials and methods, while number in the box brackets indicate the number of FW-infested fields that were detected outside of the formally surveyed transects. ^3^ Mean incidence (%) recorded in surveyed transects in FW-infested fields. Where the FW was detected outside of the surveyed transects, it was indicated as ‘Detected’. In fields where Fusarium wilt was detected, the range of disease incidence from lowest to highest is in parentheses. na: data were not available since no formal survey was conducted.

**Table 3:** Summary of number of NSW *Fusarium* isolates recovered from 2017 to 2022 from FW-suspected seedlings and mature cotton plants, and number of Fov isolates determined by specific SIX6 marker.

Valleys	Season 17/18		Season 18/19		Season 19/20		Season 20/21		Season 21/22	
	No Fusarium	No Fov	No Fusarium	No Fov	No Fusarium	No Fov	No Fusarium	No Fov	No Fusarium	No Fov
**Gwydir**	32	32	54	54	38	38	182	171	274	256
**Namoi**	3	0	0	0	2	0	2	2	10	4
**Macquarie**	18	4	28	28	0	0	0	0	33	33
**Lachlan**	0	0	0	0	36	18	4	0	0	0
**Murrumbidgee**	0	0	14	14	3	3	10	10	0	0

## Supplementary Materials

The following supporting information can be downloaded at: https://www.mdpi.com/article/10.3390/pathogens11121537/s1, Table S1: Brief summary of *Fusarium oxysporum* isolates recovered from FW-diseased cotton samples across NSW and QLD that were subjected to TEF1 sequencing, SIX6 typing and VCG analyses.
